# Trends in Taxonomy of Chagas Disease Vectors (Hemiptera, Reduviidae, Triatominae): From Linnaean to Integrative Taxonomy

**DOI:** 10.3390/pathogens10121627

**Published:** 2021-12-15

**Authors:** Kaio Cesar Chaboli Alevi, Jader de Oliveira, Dayse da Silva Rocha, Cleber Galvão

**Affiliations:** 1 Laboratório de Parasitologia, Faculdade de Ciências Farmacêuticas, Universidade Estadual Paulista “Júlio de Mesquita Filho” (UNESP), Rodovia Araraquara-Jaú km 1, Araraquara 14801-902, Brazil; kaiochaboli@hotmail.com (K.C.C.A.); jdr.oliveira@hotmail.com (J.d.O.); 2 Laboratório de Entomologia em Saúde Pública, Faculdade de Saúde Pública, Universidade de São Paulo (USP), Av. Dr. Arnaldo 715, São Paulo 01246-904, Brazil; 3 Laboratório Nacional e Internacional de Referência em Taxonomia de Triatomíneos, Instituto Oswaldo Cruz (FIOCRUZ), Av. Brasil 4365, Pavilhão Rocha Lima, Sala 505, Rio de Janeiro 21040-360, Brazil; dayseroch@gmail.com

**Keywords:** Triatominae, classical taxonomy, molecular taxonomy, integrative taxonomy

## Abstract

Chagas disease is a neglected tropical disease caused by the protozoan *Trypanosoma cruzi* and transmitted mainly by members of the subfamily Triatominae. There are currently 157 species, grouped into 18 genera and five tribes. Most descriptions of triatomine species are based on classical taxonomy. Facing evolutionary (cryptic speciation and phenotypic plasticity) and taxonomic (more than 190 synonymizations) problems, it is evident that integrative taxonomy studies are an important and necessary trend for this group of vectors. Almost two-and-a-half centuries after the description of the first species, we present for the first time the state-of-the-art taxonomy of the whole subfamily, covering from the initial classic studies to the use of integrative taxonomy.

## 1. Triatominae: The Vectors of Chagas Disease

Chagas disease is a neglected tropical disease caused by the protozoan *Trypanosoma cruzi* (Chagas, 1909) (Kinetoplastida, Trypanosomatidae) [1]. This disease is found mainly in 21 Latin American countries, where it is mostly vector-borne, more specifically by members of the subfamily Triatominae (Hemiptera, Reduviidae) [1]. Triatomines or kissing bugs are hematophagous insects that have a habit of defecating during or after the blood meal—if they are infected with *T. cruzi*, they release the parasite in the feces/urine [1]. An estimated 8 million people are infected worldwide, and more than 65 million people at risk of acquiring the disease, which causes more than 12,000 deaths per year, the vector control being the most useful method to prevent new infections [1,2].

There are currently 157 species (154 extant species and three fossils), grouped into 18 genera and five tribes (Table 1) [3,4,5,6,7], being all potential vectors of *T. cruzi*. Taxonomic studies of Triatominae started in the 18th century with the description of *Triatoma rubrofasciata* (De Geer, 1773) (as *Cimex rubro-fasciatus*) [8]. Almost two and a half centuries after the description of the first species, we presented for—the first time—a review of the state-of-the-art of taxonomy of the whole subfamily, covering from the initial classic studies to the use of integrative taxonomy, a term formally introduced only in 2005 to describe taxa by integrating information from different data and methodologies [9,10].

## 2. Applications and Limitations of Triatominae Taxonomic Studies

For 225 years (1773–1998), the descriptions of triatomine species have been based only on studies of classical taxonomy (using descriptive morphology, comparative morphology, and/or morphometry) (Table 2). Although these analyses are imperative and are present in the description of all species of the subfamily Triatominae (Table 2), in the last decade, other approaches (such as biochemical [5,11], cytogenetic [5,12], phylogenetic [5,13,14,15,16,17] and/or of reproductive barriers [5]) started to be combined with the characterization of morphology and/or morphometry, employing the integrative taxonomy in the study of these insect vectors (Table 2).

More than 190 synonymization acts occurred in the subfamily Triatominae [18,19], with the majority of synonymized taxa being described from classical taxonomy. The use of combined analyses for the characterization of a taxon greatly reduces the chances of synonymization (although it does not make it impossible [19,20]). Based on the synonymization events and the importance of multi-analyses for the characterization of a taxon, we will discuss the current issues, applications, and limitations of classical, molecular, and integrative taxonomy.

### 2.1. Classical Taxonomy

Classical taxonomy underlies most taxonomic studies of species description in the subfamily Triatominae (Table 2). The morphological and morphometric studies applied in the last described taxa are: morphological study of the head, thorax, abdomen, and male and female genitalia (with optical microscopy (OM) and/or scanning electronic microscopy (SEM)), and morphometric study of the head, thorax, abdomen and appendices (using OM) [5,6,7,15,16,17,132].

Although the use of morphological and morphometric characters is essential to describe a new taxon (since the diagnosis of the species needs to be made based on specimens that will be deposited, such as vouchers, in entomological collections), evolutionary events of cryptic speciation [14] and phenotypic plasticity [14] present in the subfamily Triatominae can make it difficult to diagnose a taxon only by morphological studies. Classic examples of this can be seen in the genus *Rhodnius* Stål, 1859: *R. montenegrensis* Rosa et al., 2012 [13] and *R. marabaensis* Souza et al., 2017 [15] represent two of the four paraphyletic strains of *R. robustus* Larrousse, 1927 [134,135] (the application of integrative taxonomy allowed description of the species from specimens initially characterized as *R. robustus* [136]). On the other hand, was demonstrated that *R. taquarussiensis* Rosa et al., 2017 (species described by integrative taxonomy [20]) represented only an intraspecific polymorphism of *R. neglectus* Lent, 1954 [19] (from studies of molecular taxonomy combined with experimental crosses it was possible to synonymize the species [19]).

Morphological convergence events can also hinder the classic taxonomy of these vectors [129]. The paraphyletic genus *Triatoma* Laporte, 1832 needs several studies from a taxonomic and systematic point of view [137]. *Triatoma tibiamaculata* (Pinto, 1926), for example, is a species that has morphological characteristics that bring it together and groups it (until now) as a *Triatoma* [138]. However, the generic status of this vector has been questioned several times [134,137,138]—since it presents cytogenetic [139], structural [140] and phylogenetic [137,138] characteristics that bring it closer to *Panstrongylus* (which highlights the importance of studies with integrative taxonomy).

### 2.2. Molecular Taxonomy

The first phylogenetic trees with molecular markers were published only in 1998 [141], giving rise to the phylogenetic systematics and molecular taxonomy of these vectors. Although no species of triatomine has been described by molecular taxonomy (Table 2), the combination of phylogenetic analyses with morphological and morphometric studies in species description studies (integrative taxonomy) has been a trend in the last decade [5,13,14,15,16,17] (Table 2), since it provides greater reliability of the specific status of the taxa and allows, above all, to understand the evolutionary history of the species.

In addition to the contributions mentioned above, molecular taxonomy and phylogenetic systematics allowed the evaluation and re-validation of the taxonomic status of some species: reinclusion of *Linshcosteus* Distant, 1904 genus in Triatomini tribe (extinguishing the Linshcosteini tribe) [30]; inclusion of *Psammolestes* Bergroth, 1911 species in the genus *Rhodnius* [30] (proposal not accepted by the scientific community due to the differences that support the generic status of *Psammolestes* [17]); inclusion of the species *T. flavida* Neiva, 1911, and *N. obscura* Maldonado & Farr, 1962 in the genus *Nesotriatoma* Usinger, 1944 [142]; confirmation of the generic status of *Nesotriatoma* [132]; inclusion of species *T. spinolai* Porter, 1934, *M. gajardoi* Frias, Henry & Gonzalez, 1998, *T. eratyrusiformis* Del Ponte, 1929, and *T. breyeri* Del Ponte, 1929 in the genus *Mepraia* Mazza, Gajardo & Jörg, 1940 [142] (partially accepted suggestion, being the *Mepraia* genus currently composed of *M. spinolai*, *M. gajardoi*, and *M. parapatrica* Frías-Lasserre, 2010 [4,143]); confirmation of the generic status of *Mepraia* [137]; and inclusion of *T. dimidiata* (Latreille, 1811) in the *Meccus* Stål, 1859 genus (genus that later was considered invalid and the *Meccus* species started to be considered as *Triatoma* [137,144,145]).

Although the International Code of Zoological Nomenclature does not consider groupings of triatomines to be complexes or subcomplexes [146], Justi et al. [137] suggests that these groupings should represent monophyletic groups. In the genus *Triatoma*, for example, studies based on phylogenetic systematics evaluated the position of several species that had been grouped mainly by geographic distribution and morphological similarities and proposed regrouping and/or the creation of new monophyletic groups [137,147,148]. Species well defined as natural groups (monophyletic) are currently the *T. brasiliensis* [149,150], *T. sordida* [151], *T. rubrovaria* [151], *T. infestans* [137], and *T. vitticeps* [148] subcomplexes.

### 2.3. Integrative Taxonomy

The data integration in the integrative taxonomy can be done by cumulation or congruence [152]. The use of combined tools to delimit a species of triatomine occurred for the first time in 1998 by Frias et al. [111] who combined morphological, morphometric, cytogenetic, and reproductive barriers data to describe *M. gajardoi* (Table 2). However, only in the last decade has the integrative taxonomy has been more applied in the study of these vectors (Table 2).

This tendency to integrate different analyses to characterize a taxon, made it possible to resolve ancient taxonomic issues, such as the description by *T. mopan* Dorn et al. (2018) and *T. huehuetenanguensis* Lima-Cordón et al. (2019) from specimens initially characterized as *T. dimidiata* [16,17,153,154] and the recent description of *T. rosai* Alevi et al., 2020 from the allopatric population of *T. sordida* (Stål, 1859) from Argentina [5,155,156]. In addition, the specific status of *T. bahiensis* Sherlock & Serafim, 1967 (a species that for more than three decades has been synonymous with *T. lenti* Sherlock & Serafim, 1967 [101]) has been revalidated based on integrative taxonomy [149].

On the other hand, even if the integrative taxonomy provides more robustness in the characterization of the new taxa (decreasing the chance of synonymization), does not prevent this event can occur (as mentioned above for *R. taquarussuensis* which has been synonymous with *R. neglectus* Lent, 1954 [19]). Although morphological, morphometric, and cytogenetic intraspecific variation had been described in the genus *Rhodnius* [157,158], the description of *R. taquarussuensis* was based on these factors [20]. Thus, synonymization event occurred through phylogenetic analyses and experimental crosses [19]. We suggest that integrative taxonomy work should include molecular studies and, whenever possible, reproductive barriers to confirm the taxon specific status following the biological concept of species [159,160,161].

In general, most articles of description based on integrative taxonomy combine only morphological and morphometric data with molecular analyses (Table 2). However, it is worth mentioning that in 2020 the description of *T. rosai* was published based on morphometric, morphological, molecular data, and experimental crosses that have been combined with information from the literature about the species (cytogenetic data [155,156], electrophoresis pattern [155], cuticular hydrocarbons pattern [162], geometric morphometry [163], cycle, and average time of life [164,165,166] as well as geographic distribution [18,42,43,44,50,51]), becoming the most complete article of species description of the subfamily Triatominae [5].

## 3. Overview of Tools Applied to Taxonomic Studies of Triatomines

In addition to species descriptions, several taxonomic studies have been carried out to assess the specific status of valid species and, above all, to assist in the correct classification of Chagas disease vectors. Based on this, we will specifically discuss the application of each taxonomic tool.

### 3.1. Morphology and Comparative Morphology

As already mentioned above, morphological studies are applied to all formal species descriptions (Table 2). These analyses can characterize several structures that, in general, are compared and confirm the specific status of triatomines [5,6,11,12,13,14,15,16,17]. Studies with OM and SEM allow characterizing structures of the head, thorax, and abdomen. These analyses are very important for classical taxonomy and support the main dichotomous keys used for the correct identification of these vectors [101,167,168,169,170,171,172].

### 3.2. Morphometry

Like morphological studies, morphometric studies are also present in the description of all triatomines (at first, showing the size of specimens and structures and, later, by means of geometric morphometry [4]). These measurable data are very important from a taxonomic point of view, as a visual identification system was recently developed from morphometric data that has the potential to automate the identification of triatomines [173,174].

### 3.3. Chemotaxonomy

In 1964, Actis et al. [175] used, for the first time, biochemical studies with hemolymph protein electrophoresis to compare species of triatomines, giving rise to chemotaxonomy. Isoenzymes were applied to different species of *Rhodnius* [176], the *T. brasiliensis* subcomplex [177] and Mexican *Triatoma* [178]. However, recently, biochemical studies are rare from a taxonomic perspective; they contribute to the integrative taxonomy as shown by Jurberg et al. [11] and Alevi et al. [5] with the species descriptions of *T. pintodiasi* Jurberg et al., 2013 and *T. rosai* respectively.

### 3.4. Cytotaxonomy and Karyosystematic

Cytotaxonomy was started with Ueshima [179] by proposing the application of cytogenetic studies of chromosomes to differentiate morphologically related species. Later, the use of chromosomal analyses—such as karyotypes [180,181,182,183]—the constitutive heterochromatin pattern [156,184,185], the heterochromatin base pair composition [186,187,188], and the location of the nucleolar organizing region [139,156,189], assisted in the correct identification and classification of triatomines. Recently, dichotomous keys have been proposed based on cytogenetic data [190,191,192,193].

### 3.5. MALDI-TOF MS

Laroche et al. [194] used, for the first time, matrix-assisted laser desorption/ionization time-of-flight mass spectrometry (MALDI-TOF MS) analysis to differentiate triatomine species. The researchers were able to differentiate species from French Guiana by MALDI-TOF. Subsequently, Souza et al. [195] used these analyses to differentiate 12 species of the genus Rhodnius. Furthermore, Souza et al. [196] also differentiated the species of Cavernicola Barber, 1937.

### 3.6. Omics

In 2017, omics tools (transcriptomics) were used for the first time in taxonomic studies of triatomines to confirm the specific status of *R. montenegrensis* [197]. In 2019, Brito et al. [198] also validated the specific status of *R. montenegrensis* and confirmed that this species refers to strain II of the paraphyletic group of *R. robustus*.

## 4. Concluding Remarks

**Figure 1:**
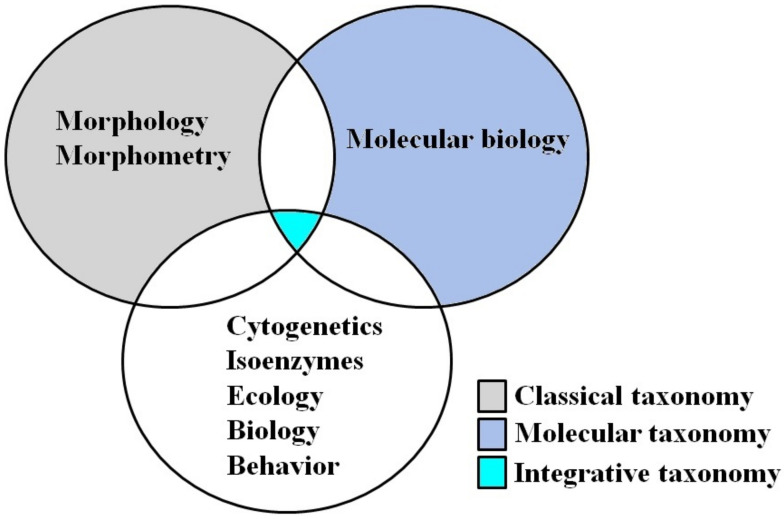
Schematic representation of the integrative taxonomy of triatomines.

Classical taxonomy, over the last few decades, has been revitalized by integrative taxonomy leading to success in the identification and delimitation of new species through the use of multiple and complementary approaches. Most descriptions of triatomine species are based on classical taxonomy. Facing evolutionary (cryptic speciation and phenotypic plasticity) and taxonomic (more than 190 synonymizations) problems has indicated that it is evident that integrative taxonomy studies are an important and necessary trend for this group of vectors. However, from the synonymization of *R. taquarussuensis* (which was described through integrative taxonomy [20] and was later synonymized with *R. neglectus* [19]), it is evident that phylogenetic studies (molecular taxonomy) should be considered among the analyses used for the description of new species from the integrative taxonomy (Figure 1).

## Figures and Tables

**Table 1:** Tribes, genera, and number of species that make up the subfamily Triatominae.

Tribe	Genus	Species (n)
Alberproseniini	*Alberprosenia*	2
Bolboderini	*Belminus*	9
	*Bolbodera*	1
	*Microtriatoma*	2
	*Parabelminus*	2
Cavernicolini	*Cavernicola*	2
Rhodniini	*Psammolestes*	3
	*Rhodnius*	21
Triatomini	*Dipetalogaster*	1
	*Eratyrus*	2
	*Hermanlentia*	1
	*Linshcosteus*	6
	*Mepraia*	3
	*Nesotriatoma*	3
	*Panstrongylus*	15
	*Paratriatoma*	2
	*Triatoma*	81
	*Paleotriatoma*	1
Total		157

**Table 2:** Species, taxonomic tools, and taxonomic classification used in the description of Triatominae taxa.

	Species	Morphology and Morphometry	Chemotaxonomy	Cytotaxonomy	Experimental Crosses	Phylogenetic Systematics and Molecular Taxonomy	Taxonomy	References
**1**	*Triatoma rubrofasciata* (De Geer, 1773)	**X**					Classical taxonomy	De Geer [8]
**2**	*Triatoma dimidiata* (Latreille, 1811)	**X**					Classical taxonomy	Latreille [21]
**3**	*Panstrongylus geniculatus* (Latreille, 1811)	**X**					Classical taxonomy	Latreille [21]
**4**	*Triatoma infestans* (Klug, 1834)	**X**					Classical taxonomy	Klug [22]
**5**	*Triatoma phyllosomus* (Burmeister, 1835)	**X**					Classical taxonomy	Burmeister [23]
**6**	*Panstrongylus megistus* (Burmeister, 1835)	**X**					Classical taxonomy	Burmeister [23]
**7**	*Triatoma rubrovaria* (Blanchard, 1846)	**X**					Classical taxonomy	Blanchard [24]
**8**	*Triatoma maculata* (Erichson, 1848)	**X**					Classical taxonomy	Erichson [25]
**9**	*Triatoma mexicana* (Herrich-Schaeffer, 1848)	**X**					Classical taxonomy	Herrich-Schaeffer [26]
**10**	*Triatoma sanguisuga* (Leconte, 1855)	**X**					Classical taxonomy	Leconte [27]
**11**	*Belminus rugulosus* (Stål, 1859)	**X**					Classical taxonomy	Stål [28]
**12**	*Eratyruscuspidatus* (Stål, 1859)	**X**					Classical taxonomy	Stål [28]
**13**	*Eratyrusmucronatus* (Stål, 1859)	**X**					Classical taxonomy	Stål [28]
**14**	*Rhodnius nasutus* (Stål, 1859)	**X**					Classical taxonomy	Stål [28]
**15**	*Rhodnius prolixus* (Stål, 1859)	**X**					Classical taxonomy	Stål [28]
**16**	*Triatoma circummaculata* (Stål, 1859)	**X**					Classical taxonomy	Stål [28]
**17**	*Triatoma gerstaeckeri* (Stål, 1859)	**X**					Classical taxonomy	Stål [28]
**18**	*Paratriatoma lecticularia* (Stål, 1859)	**X**					Classical taxonomy	Stål [28]
**19**	*Triatoma sordida* (Stål, 1859)	**X**					Classical taxonomy	Stål [28]
**20**	*Triatoma vitticeps* (Stål, 1859)	**X**					Classical taxonomy	Stål [28]
**21**	*Triatoma recurva* (Stål, 1868)	**X**					Classical taxonomy	Stål [29]
**22**	*Triatoma venosa* (Stål, 1872)	**X**					Classical taxonomy	Stål [30]
**23**	*Triatoma pallidipennis* (Stål, 1872)	**X**					Classical taxonomy	Stål [30]
**24**	*Rhodnius pictipes* (Stål, 1872)	**X**					Classical taxonomy	Stål [30]
**25**	*Triatoma nigromaculata* (Stål, 1872)	**X**					Classical taxonomy	Stål [30]
**26**	*Panstrongylus lignarius* (Walker, 1873)	**X**					Classical taxonomy	Walker [31]
**27**	*Panstrongylus guentheri* (Berg, 1879)	**X**					Classical taxonomy	Berg [32]
**28**	*Triatoma rubida* (Uhler, 1894)	**X**					Classical taxonomy	Uhler [33]
**29**	*Dipetalogaster maxima* (Uhler, 1894)	**X**					Classical taxonomy	Uhler [33]
**30**	*Triatoma protracta* (Uhler, 1894)	**X**					Classical taxonomy	Uhler [33]
**31**	*Panstrongylus rufotuberculatus* (Champion, 1899)	**X**					Classical taxonomy	Champion [34]
**32**	*Triatoma migrans* (Breddin, 1903)	**X**					Classical taxonomy	Breddin [35]
**33**	*Linshcosteuscarnifex* (Distant, 1904)	**X**					Classical taxonomy	Distant [36]
**34**	*Bolbodera scabrosa* (Valdés, 1910)	**X**					Classical taxonomy	Valdés [37]
**35**	*Nesotriatoma flavida* (Neiva, 1911)	**X**					Classical taxonomy	Neiva [38]
**36**	*Psammolestes coreodes* (Bergroth, 1911)	**X**					Classical taxonomy	Bergroth [39]
**37**	*Panstrongylus howardi* (Neiva, 1911)	**X**					Classical taxonomy	Neiva [40]
**38**	*Triatoma brasiliensis* (Neiva, 1911)	**X**					Classical taxonomy	Neiva [41]
**39**	*Triatoma neotomae* (Neiva, 1911)	**X**					Classical taxonomy	Neiva [42]
**40**	*Triatoma indictiva* (Neiva, 1912)	**X**					Classical taxonomy	Neiva [43]
**41**	*Triatoma platensis* (Neiva, 1913)	**X**					Classical taxonomy	Neiva [44]
**42**	*Rhodnius brethesi* (Matta, 1919)	**X**					Classical taxonomy	Matta [45]
**43**	*Panstrongylus lutzi* (Neiva & Pinto, 1923)	**X**					Classical taxonomy	Neiva and Pinto [46]
**44**	*Rhodnius domesticus* (Neiva & Pinto, 1923)	**X**					Classical taxonomy	Neiva and Pinto [47]
**45**	*Triatoma melanocephala* (Neiva & Pinto, 1923)	**X**					Classical taxonomy	Neiva and Pinto [48]
**46**	*Triatoma bouvieri* (Larrousse, 1924)	**X**					Classical taxonomy	Larrousse [49]
**47**	*Triatoma petrocchiae* (Pinto & Barreto, 1925)	**X**					Classical taxonomy	Pinto and Barreto [50]
**48**	*Psammolestes arthuri* (Pinto, 1926)	**X**					Classical taxonomy	Pinto [51]
**49**	*Triatoma carrioni* (Larrousse, 1926)	**X**					Classical taxonomy	Larrousse [52]
**50**	*Triatoma tibiamaculata* (Pinto, 1926)	**X**					Classical taxonomy	Pinto [53]
**51**	*Rhodnius robustus* (Larrousse, 1927)	**X**					Classical taxonomy	Larrousse [54]
**52**	*Panstrongylus chinai* (Del Ponte, 1929)	**X**					Classical taxonomy	Del Ponte [55]
**53**	*Triatoma breyeri* (Del Ponte, 1929)	**X**					Classical taxonomy	Del Ponte [55]
**54**	*Triatoma eratyrusiformis* (Del Ponte, 1929)	**X**					Classical taxonomy	Del Ponte [55]
**55**	*Triatoma limai* (Del Ponte, 1929)	**X**					Classical taxonomy	Del Ponte [55]
**56**	*Triatoma patagonica* (Del Ponte, 1929)	**X**					Classical taxonomy	Del Ponte [55]
**57**	*Rhodnius pallescens* (Barber, 1932)	**X**					Classical taxonomy	Barber [56]
**58**	*Triatoma leopoldi* (Schoudeten, 1933)	**X**					Classical taxonomy	Schoudeten [57]
**59**	*Mepraia spinolai* (Porter, 1934)	**X**					Classical taxonomy	Porter [58]
**60**	*Cavernicola pilosa* (Barber, 1937)	**X**					Classical taxonomy	Barber [59]
**61**	*Paratriatoma hirsuta* (Barber, 1938)	**X**					Classical taxonomy	Barber [60]
**62**	*Triatoma longipennis* (Usinger, 1939)	**X**					Classical taxonomy	Usinger [61]
**63**	*Triatoma picturatus* (Usinger, 1939)	**X**					Classical taxonomy	Usinger [61]
**64**	*Panstrongylus humeralis* (Usinger, 1939)	**X**					Classical taxonomy	Usinger [61]
**65**	*Triatoma barberi* (Usinger, 1939)	**X**					Classical taxonomy	Usinger [61]
**66**	*Triatoma incrassata* (Usinger, 1939)	**X**					Classical taxonomy	Usinger [61]
**67**	*Triatoma nitida* (Usinger, 1939)	**X**					Classical taxonomy	Usinger [61]
**68**	*Triatoma oliveirai* (Neiva et al., 1939)	**X**					Classical taxonomy	Neiva et al. [62]
**69**	*Triatoma arthurneivai* (Lent & Martins, 1940)	**X**					Classical taxonomy	Lent and Martins [63]
**70**	*Triatoma hegneri* (Mazzotti, 1940)	**X**					Classical taxonomy	Mazzotti [64]
**71**	*Triatoma peninsularis* (Usinger, 1940)	**X**					Classical taxonomy	Usinger [65]
**72**	*Triatoma mazzottii* (Usinger, 1941)	**X**					Classical taxonomy	Usinger [66]
**73**	*Triatoma melanica* (Neiva & Lent, 1941)	**X**					Classical taxonomy	Neiva and Lent [67]
**74**	*Panstrongylus tupynambai* (Lent, 1942)	**X**					Classical taxonomy	Lent [68]
**75**	*Parabelminus carioca* (Lent, 1943)	**X**					Classical taxonomy	Lent [69]
**76**	*Panstrongylus diasi* (Pinto & Lent, 1946)	**X**					Classical taxonomy	Pinto and Lent [70]
**77**	*Triatoma delpontei* (Romaña & Abalos, 1947)	**X**					Classical taxonomy	Romaña and Abalos [71]
**78**	*Triatoma guasayana* (Wygodzinsky & Abalos, 1949)	**X**					Classical taxonomy	Wygodzinsky and Abalos [72]
**79**	*Triatoma dispar* (Lent, 1950)	**X**					Classical taxonomy	Lent [73]
**80**	*Triatoma wygodzinskyi* (Lent, 1951a)	**X**					Classical taxonomy	Lent [74]
**81**	*Microtriatoma trinidadensis* (Lent, 1951b)	**X**					Classical taxonomy	Lent [75]
**82**	*Triatoma amicitiae* (Lent, 1951c)	**X**					Classical taxonomy	Lent [76]
**83**	*Rhodnius neivai* (Lent, 1953)	**X**					Classical taxonomy	Lent [77]
**84**	*Triatoma matogrossensis* (Leite & Barbosa, 1953)	**X**					Classical taxonomy	Leite and Barbosa [78]
**85**	*Triatoma pugasi* (Lent, 1953b)	**X**					Classical taxonomy	Lent [79]
**86**	*Rhodnius neglectus* (Lent, 1954)	**X**					Classical taxonomy	Lent [80]
**87**	*Belminus costaricencis* (Herrer et al., 1954)	**X**					Classical taxonomy	Herrer et al. [81]
**88**	*Belminus peruvianus* (Herrer et al., 1954)	**X**					Classical taxonomy	Herrer et al. [81]
**89**	*Rhodnius ecuadoriensis* (Lent & León, 1958)	**X**					Classical taxonomy	Lent and León [82]
**90**	*Triatoma costalimai* (Verano & Galvão, 1958)	**X**					Classical taxonomy	Verano and Galvão [83]
**91**	*Nesotriatoma obscura* (Maldonado & Farr, 1962)	**X**					Classical taxonomy	Maldonado and Farr [84]
**92**	*Triatoma sinaloensis* (Ryckman, 1962)	**X**					Classical taxonomy	Ryckman [85]
**93**	*Triatoma pseudomaculata* (Corrêa & Espínola, 1964)	**X**					Classical taxonomy	Corrêa and Espínola [86]
**94**	*Psammolestes tertius* (Lent & Jurberg, 1965)	**X**					Classical taxonomy	Lent and Jurberg [87]
**95**	*Triatoma sinica* (Hsiao, 1965)	**X**					Classical taxonomy	Hsiao [88]
**96**	*Triatoma williami* (Galvão et al., 1965)	**X**					Classical taxonomy	Galvão et al. [89]
**97**	*Triatoma bahiensis* (Sherlock & Serafim, 1967)	**X**					Classical taxonomy	Sherlock and Serafim [90]
**98**	*Triatoma deaneorum* (Galvão et al., 1967)	**X**					Classical taxonomy	Galvão et al. [91]
**99**	*Triatoma garciabesi* (Carcavallo et al., 1967)	**X**					Classical taxonomy	Carcavallo et al. [92]
**100**	*Triatoma lenti* (Sherlock & Serafim, 1967)	**X**					Classical taxonomy	Sherlock and Serafim [90]
**101**	*Panstrongylus lenti* (Galvão & Palma, 1968)	**X**					Classical taxonomy	Galvão and Palma [93]
**102**	*Triatoma ryckmani* (Zeledón & Ponce, 1972)	**X**					Classical taxonomy	Zeledón and Ponce [94]
**103**	*Rhodnius amazonicus* (Almeida et al., 1973)	**X**					Classical taxonomy	Almeida et al. [95]
**104**	*Linshcosteusconfumus* (Ghauri, 1976)	**X**					Classical taxonomy	Ghauri [96]
**105**	*Linshcosteuscostalis* (Ghauri, 1976)	**X**					Classical taxonomy	Ghauri [96]
**106**	*Rhodnius dalessandroi* (Carcavallo & Barreto, 1976)	**X**					Classical taxonomy	Carcavallo and Barreto [97]
**107**	*Alberproseniagoyovargasi* (Martínez & Carcavallo, 1977)	**X**					Classical taxonomy	Martínez and Carcavallo [98]
**108**	*Rhodnius paraensis* (Sherlock et al., 1977)	**X**					Classical taxonomy	Sherlock et al. [99]
**109**	*Triatoma cavernicola* (Else & Cheong, 1977)	**X**					Classical taxonomy	Else et al. [100]
**110**	*Belminus herreri* (Lent & Wygodzinsky, 1979)	**X**					Classical taxonomy	Lent and Wygodzinsky [101]
**111**	*Linshcosteuschota* (Lent & Wygodzinsky, 1979)	**X**					Classical taxonomy	Lent and Wygodzinsky [101]
**112**	*Linshcosteuskali* (Lent & Wygodzinsky, 1979)	**X**					Classical taxonomy	Lent and Wygodzinsky [101]
**113**	*Microtriatoma borbai* (Lent & Wygodzinsky, 1979)	**X**					Classical taxonomy	Lent and Wygodzinsky [101]
**114**	*Parabelminus yurupucu* (Lent & Wygodzinsky, 1979)	**X**					Classical taxonomy	Lent and Wygodzinsky [101]
**115**	*Triatoma guazu* (Lent & Wygodzinsky, 1979)	**X**					Classical taxonomy	Lent and Wygodzinsky [101]
**116**	*Alberproseniamalheiroi* (Serra et al., 1980)	**X**					Classical taxonomy	Serra et al. [102]
**117**	*Triatoma brailovskyi* (Martínez et al., 1984)	**X**					Classical taxonomy	Martínez et al. [103]
**118**	*Cavernicola lenti* (Barrett & Arias, 1985)	**X**					Classical taxonomy	Barrett and Arias [104]
**119**	*Triatoma bolivari* (Carcavallo et al., 1987)	**X**					Classical taxonomy	Carcavallo et al. [105]
**120**	*Hermanlentia matsunoi* (Fernández-Loayza, 1989)	**X**					Classical taxonomy	Fernández-Loayza [106]
**121**	*Rhodnius stali* (Lent et al., 1993)	**X**					Classical taxonomy	Lent et al. [107]
**122**	*Belminus pittieri* (Osuna & Ayala, 1993)	**X**					Classical taxonomy	Osuna and Ayala [108]
**123**	*Triatoma gomeznunezi* (Martínez et al., 1994)	**X**					Classical taxonomy	Martínez et al. [109]
**124**	*Belminus laportei* (Lent et al., 1995)	**X**					Classical taxonomy	Lent et al. [110]
**125**	*Mepraia gajardoi* (Frias et al., 1998)	**X**		**X**	**X**		Integrative taxonomy	Frias et al. [111]
**126**	*Triatoma carcavalloi* (Jurberg et al., 1998)	**X**					Classical taxonomy	Jurberg et al. [112]
**127**	*Triatoma jurbergi* (Carcavallo et al., 1998)	**X**					Classical taxonomy	Carcavallo et al. [113]
**128**	*Triatoma bassolsae* (Alejandre Aguilar et al., 1999)	**X**					Classical taxonomy	Aguilar et al. [114]
**129**	*Rhodnius colombiensis* (Mejia et al., 1999)	**X**					Classical taxonomy	Mejia et al. [115]
**130**	*Triatoma baratai* (Carcavallo & Jurberg, 2000)	**X**					Classical taxonomy	Carcavallo and Jurberg [116]
**131**	*Rhodnius milesi* (Carcavallo et al., 2001)	**X**					Classical taxonomy	Valente et al. [117]
**132**	*Triatoma klugi* (Carcavallo et al., 2001)	**X**					Classical taxonomy	Carcavallo et al. [118]
**133**	*Linshcosteuskarupus* (Galvão et al., 2002)	**X**					Classical taxonomy	Galvão et al. [119]
**134**	*Triatoma sherlocki* (Papa et al., 2002)	**X**					Classical taxonomy	Papa et al. [120]
**135**	*Triatoma vandae* (Carcavallo et al., 2002)	**X**					Classical taxonomy	Carcavallo [121]
**136**	*Triatoma dominicana* (Ponair Jr., 2005)	**X**					Classical taxonomy	Ponair Jr. [122]
**137**	*Belminus corredori* (Galvão & Angulo, 2006)	**X**					Classical taxonomy	Galvão and Ângulo [123]
**138**	*Belminus ferroae* (Sandoval et al., 2007)	**X**					Classical taxonomy	Sandoval et al. [124]
**139**	*Panstrongylus mitarakaensis* (Bérenger & Blanchet, 2007)	**X**					Classical taxonomy	Bérenger and Blanchet [125]
**140**	*Triatoma boliviana* (Martinez et al., 2007)	**X**					Classical taxonomy	Martinez et al. [126]
**141**	*Triatoma juazeirensis* (Costa & Felix, 2007)	**X**					Classical taxonomy	Costa and Felix [127]
**142**	*Panstrongylus martinezorum* (Ayala, 2009)	**X**					Classical taxonomy	Ayala [128]
**143**	*Rhodnius zeledoni* (Jurberg et al., 2009)	**X**					Classical taxonomy	Jurberg et al. [129]
**144**	*Mepraia parapatrica* (Frías-Lasserre, 2010)	**X**		**X**			Integrative taxonomy	Frías-Lasserre [12]
**145**	*Rhodnius montenegrensis* (Rosa et al., 2012)	**X**				**X**	Integrative taxonomy	Rosa et al. [13]
**146**	*Panstrongylus hispaniolae* (Ponair Jr., 2013)	**X**					Classical taxonomy	Ponair Jr. [130]
**147**	*Rhodnius barretti* (Abad-Franch et al., 2013)	**X**				**X**	Integrative taxonomy	Abad-Franch et al. [14]
**148**	*Triatoma jatai* (Gonçalves et al., 2013)	**X**					Classical taxonomy	Gonçalves et al. [131]
**149**	*Triatoma pintodiasi* (Jurberg et al., 2013)	**X**	**X**				Integrative taxonomy	Jurberg et al. [11]
**150**	*Rhodnius marabaensis* (Souza et al., 2017)	**X**				**X**	Integrative taxonomy	Souza et al. [15]
**151**	*Nesotriatoma confusa* (Oliveira et al., 2018)	**X**					Classical taxonomy	Oliveira et al. [132]
**152**	*Triatoma mopan* (Dorn et al., 2018)	**X**				**X**	Integrative taxonomy	Dorn et al. [16]
**153**	*Paleotriatoma metaxytaxa* (Poinar Jr., 2019 )	**X**					Classical taxonomy	Poinar Jr. [133]
**154**	*Triatoma huehuetenanguensis* (Lima-Cordon et al., 2019)	**X**				**X**	Integrative taxonomy	Lima-Cordon et al. [17]
**155**	*Triatoma rosai* (Alevi et al., 2020)	**X**	**X**	**X**	**X**	**X**	Integrative taxonomy	Alevi et al. [5]
**156**	*Rhodnius micki* (Zhao et al., 2021)	**X**					Classical taxonomy	Zhao et al. [6]
**157**	*Belminus santosmalletae* (Dale et al., 2021)	**X**					Classical taxonomy	Dale et al. [7]

## Data Availability

All relevant data are within the manuscript.
